# What’s all the talk about? Topic modelling in a mental health Internet support group

**DOI:** 10.1186/s12888-016-1073-5

**Published:** 2016-10-28

**Authors:** Bradley Carron-Arthur, Julia Reynolds, Kylie Bennett, Anthony Bennett, Kathleen M. Griffiths

**Affiliations:** Centre for Mental Health Research, The Australian National University, 63 Eggleston Road, Acton, Canberra, ACT 2601 Australia

**Keywords:** Internet support group, Mental health, Topic modelling, Latent Dirichlet Allocation, Super users, Peer-support

## Abstract

**Background:**

The majority of content in an Internet Support Group (ISG) is contributed by 1 % of the users (‘super users’). Computational methods, such as topic modelling, can provide a large-scale quantitative objective description of this content. Such methods may provide a new perspective on the nature of engagement on ISGs including the role of super users and their possible effect on other users.

**Methods:**

A topic model was computed for all posts (*N* = 131,004) in the ISG BlueBoard using Latent Dirichlet Allocation. A model containing 25 topics was selected on the basis of intelligibility as determined by diagnostic metrics and qualitative investigation. This model yielded 21 substantive topics for further analysis. Two chi-square tests were conducted separately for each topic to ascertain: (i) if the odds of super users’ and other users’ posting differed for each topic; and (ii) if for super users the odds of posting differed depending on whether the response was to a super user or to another user.

**Results:**

The 21 substantive topics covered a range of issues related to mental health and peer-support. There were significantly higher odds that super users wrote content on 13 topics, with the greatest effects being for Parenting Role (OR [95%CI] = 7.97 [7.85–8.10]), Co-created Fiction (4.22 [4.17–4.27]), Mental Illness (3.13 [3.11–3.16]) and Positive Change (2.82 [2.79–2.84]). There were significantly lower odds for super users on 7 topics, with the greatest effects being for the topics Depression (OR = 0.27 [0.27–0.28]), Medication (0.36 [0.36–0.37]), Therapy (0.55 [0.54–0.55]) and Anxiety (0.55 [0.55–0.55]). However, super users were significantly more likely to write content on 5 out of these 7 topics when responding to other users than when responding to fellow super users.

**Conclusions:**

The findings suggest that super users serve the role of emotionally supportive companions with a focus on topics broadly resembling the consumer/carer model of recovery. Other users engage in topics with a greater focus on experiential knowledge, disclosure and informational support, a pattern resembling the clinical symptom-focussed approach to recovery. However, super users modify their content in response to other users in a manner consistent with being ‘active help providers’.

## Background

Online peer-to-peer communication is a popular source of health information and support. Recent research on Internet users in the USA found that 18 % of people had used the Internet in the last year to find information from a peer with similar health concerns [1]. Furthermore, 8 % of all Internet users had engaged in peer-support by either posting a question or sharing information based on their personal health experience [2]. Mental health concerns are a major component of this health information seeking, with 28 % of all Internet users having sought mental health information online [3].

Given this popularity, there has been interest in determining whether Internet support groups (ISGs) are effective in reducing depressive symptoms. A systematic review of ISGs encompassing all types of health conditions failed to find convincing evidence that online peer-to-peer support was associated with a reduction in depressive symptoms [4]. Moreover, a review of depression ISGs specifically reported that there was a paucity of evidence concerning the effectiveness of depression Internet support groups for symptom reduction [5]. More recently, a randomised controlled trial of a depression ISG has provided high quality evidence of depressive symptom reduction [6]. However, further research is required before firm conclusions can be drawn about the effectiveness of depression ISGs. It has been suggested that mental health internet support groups (MHISGs) increase the user’s sense of empowerment [7] and such support groups are widely used in conjunction with other psychoeducational and therapeutic Internet interventions with the aim of promoting engagement [8]. More recently, a randomised controlled trial demonstrated that a depression ISG was associated with increased empowerment, self-esteem and perceived quality of life relative to a control condition [9].

Complementary to work focused on the effectiveness of MHISGs, another stream of research has been concerned with understanding the nature of ISGs [10]. Such understanding is vital to informing practice and policy to promote the growth and sustainability of ISGs [11]. Research on the nature of these peer-to-peer groups is also needed to identify what elements of the groups are responsible for fostering user empowerment, and what components might be enhanced to increase the potential effectiveness of ISGs for symptom reduction. In a series of studies on the nature of the Australian ISG BlueBoard, we have so far investigated the distribution of user engagement across the ISG [12], characteristics of users which predict user engagement and retention (Griffiths KM, Carron-Arthur B, Reynolds J, Bennett K, Bennett A: User characteristics and usage of an open access moderated Internet support group for depression and other mental disorders: A prospective study, Submitted) and the community structure of the ISG [13]. This research has shown that more highly engaged users: post vastly more than their peers in a distribution that follows Zipf’s law (inversely proportional relationship between rank and frequency) [12]; tend to be consumers rather than carers (Griffiths KM, Carron-Arthur B, Reynolds J, Bennett K, Bennett A: User characteristics and usage of an open access moderated Internet support group for depression and other mental disorders: A prospective study, Submitted); tend not to be less than 20 years old (Griffiths KM, Carron-Arthur B, Reynolds J, Bennett K, Bennett A: User characteristics and usage of an open access moderated Internet support group for depression and other mental disorders: A prospective study, Submitted); and join earlier than the peers with whom they most often communicate, leading to the formation of sub-communities within the MHISG [13]. This research has highlighted the importance of peer-leaders who are highly engaged and who communicate with many other users. These findings are also consistent those from a previous survey of MHISG users which found that highly-engaged users identify themselves as ‘active help providers’ [14]. Based on a content analysis of user posts, it has been found that highly active users provide higher levels of social support than other users in the MHISG [15]. If social support underpins improvements in outcomes among users of ISGs, the highly engaged user is likely to be an important contributor to the effectiveness of ISGs.

In a systematic review of studies investigating participation styles in online health communities, we found that the peer-leader phenomenon has been measured in a number of different ways in the literature [16]. However, the role of the peer leader has been most commonly operationalised as high posting frequency. For example, the top 1 % of users, labelled “superusers”, have been observed to contribute around 75 % of all posts in the ISG [12, 17, 18]. In our review we noted that studies commonly attributed high value to the contributions of high-posting users despite the fact that a priori, posting frequency does not in of itself necessarily contribute value to the community [16].

In an attempt to develop a more nuanced index of post frequency that factored in post quality, Preece [19] recommended counting only posts which were “on-topic”. This measure would appear to be preferable to an unadjusted frequency count. However, it assumes that posts can be validly dichotomised into ‘on-’ and ‘off-’ topic, a premise which is questionable in a mental health ISG where each person’s lived experience and needs can vary, and the relevance of a post will depend on the perspective of the reader. It may be more helpful to conceptualise posts as being relevant to varying numbers of people and to measure how many people engage in each of the various topics. More particularly, given the large number of posts created by super users, there may be value in investigating if and how the topics and frequency with which they are discussed differ between super users and other users of the ISG, as well as comparing the responses of super users to fellow super users with their responses to other users. Identifying similarities and differences in the degree to which various topics are discussed by super users and others may indicate if the majority of post content is aligned with the interests of the majority of users. This may provide an indication of the role that super users are performing with respect to supporting other users. Thus, rather than asking, “What is a more accurate measure of peer-leadership than posting frequency”, it may be more informative to ask, “Of what topics is the volume of posts in a mental health ISG comprised?” and, “How and in what circumstances does the frequency with which different topics are discussed vary between super users and other users?”.

### Content analysis

To date, most studies of the content of MHISGs have used human judges and pre-formulated coding schemes to manually classify different types of peer-support. A review of depression ISGs conducted in 2009 found that all eight of the studies which analysed content had used this method [10]. This includes typologies such as the Cutrona Support Behavior Code [20] and other systems [21, 22] which although differing somewhat generally involved common categories such as: disclosure (revealing personal information about oneself); emotional support (showing empathy and concern, and offering affection and encouragement); companionship (engaging in activities with a person to communicate a sense of shared belonging); information support (providing helpful information); and cognitive guidance (advice, offering a new perspective from which to think about an issue).

In contrast to the manual methods outlined above, computer-aided methods have provided new ways of processing linguistic content which enable themes to be automatically and objectively detected in text on a large scale. One method which has been widely used is Latent Dirichlet Allocation (LDA) [23]. LDA is a topic modelling algorithm which determines latent topics across a corpus of text based on the distribution of words across the documents which make up the whole corpus. Words which co-occur in individual documents frequently across all the documents of the corpus are allocated to categories which represent a latent topic. LDA is an unsupervised machine learning method in which the algorithm derives the topics without using a training dataset. This contrasts with supervised machine learning in which an analyst teaches the algorithm to classify particular content. The two methods contribute different utility, with unsupervised methods such as LDA being particularly useful where the analyst is seeking to generate a summary of the data that is unbiased by human input, although the analyst may adjust some parameters such as the number of topics to be discerned. The output, referred to as *topics*, takes the form of groups of words ordered by their probability of occurring in that topic. The algorithm also computes the proportion of each document that is made up of each topic.

Several previous studies have used LDA to analyse MHISGs [24, 25] and peer-to-peer conversations about mental health in other online communities such as Twitter [26], Facebook [27] or other blogging sites [28]. These studies have shown that automated algorithms can be used to differentiate between the content of mental health specific communities and the content of other ISGs or online conversations [24, 26]. Furthermore, studies have successfully used the metrics obtained from these methods to make predictions about the diagnosis or symptom severity of users [27, 28]. One study [25] showed that it was possible to detect significant differences between the written content of highly-socially-connected users and other less-connected users. However, these studies were focussed on demonstrating the capability of the tools to differentiate between users. They did not analyse the nature of those differences in a manner that might increase our understanding of the social dynamics in a MHISG.

In the current study, we aimed to ascertain the predominant topics of discussion and investigate the nature of differences in content produced by super users and other users of a MHISG. Our objective was to compare quantitatively the differences in frequency with which these groups of users write about various topics (as determined by LDA). We sought to compare the difference between the two groups as well as the difference between the responses made by super users in threads initiated by fellow super users compared with their response to threads initiated by other users.

## Method

### Data

Data for the current study was drawn from the log data of the Internet support group BlueBoard (blueboard.anu.edu.au). The dataset has been described previously [12, 13, (Griffiths KM, Carron-Arthur B, Reynolds J, Bennett K, Bennett A: User characteristics and usage of an open access moderated Internet support group for depression and other mental disorders: A prospective study, Submitted)]. Briefly, the data covered the period 1 October 2008 to 23 May 2014, in which 131,004 posts were made by 2932 users.

### The Internet support group: BlueBoard

This service was provided by the Centre for Mental Health Research at The Australian National University. BlueBoard comprised 10 forums in which users communicated about a range of mental health issues, including: eight condition specific forums (in order of usage - depression, bipolar disorder, generalized anxiety, social anxiety, borderline personality disorder, eating disorder, panic disorder and obsessive compulsive disorder); one forum dedicated for carers of people with mental health issues; and one forum for general discussion. BlueBoard was first established in 2003 as a mood disorder group. It was closed in 2007 and 2008 due to lack of funding and re-established on 1 October 2008, but closed again on 30 June 2016. This second iteration of BlueBoard, from which the current data is drawn, did not include the content or registrations of previous users. Although BlueBoard provided a service, it was also designed with the aim of being used for research purposes. Moderators did not actively participate in any of the forums. Rather they enforced rules, for example, by editing posts to remove any personally identifying information, and alerting the infracting user via a private notification. Thus the data comprises content which is solely authored by BlueBoard users. BlueBoard otherwise includes features similar to other Internet support groups such as the ability to quote other users in posts and the provision user information (total posts and registration date) displayed beside the username of each post’s author.

### Analysis

Our analysis entailed two parts. In Part 1, a computer-aided content analysis was implemented using LDA in order to model the predominant topics in BlueBoard post content. The output of this analysis included word lists which represented each of the identified topics as well as the proportion of words in each of the 131,004 posts that were from each topic. We converted these proportions to word frequencies in order to differentiate between posts with equal proportions but different word counts. In Part 2 of the analysis, we determined the degree to which the content of super users differed from that of other users in each of these topic categories.

### Part 1: Identification of topics and sentiment

The LDA analysis [23] to model topics was implemented using Mallet software with hyperparameter optimization enabled [29]. The analysis was conducted using each post as a separate document over which the distribution of topics was to be determined. The standard Mallet stop-word list containing very common English words was used to exclude such words from the analysis. Additionally, 18 contractions of common words (e.g. you’re) and the words ‘thing’ and ‘things’ were excluded.

Our objective in developing a topic model was to provide an intelligible representation of the type of content BlueBoard comprises. We therefore sought to model topics in the corpus at a level of granularity that was neither too broad nor too specific. Accordingly, selection of an appropriate number of topics was carried out by author BC following a method developed for this purpose by Evans [30]. We implemented the LDA analysis iteratively, modelling between 10 and 100 topics in increments of 10.

In the first of three phases of the analysis, we investigated the intelligibility of these models based on qualitative investigation of the word lists comprising each topic. In models containing more than 30 topics, we observed some duplications of topics where the meaning of the keywords was overly similar. For example, in the model containing 40 topics, the topic Livelihood split into two topics with top ranked words “Money, time, pay, day” in one and “work, job, week, working” in the other. This degree of redundancy in a model with more topics was judged to be less intelligible. Below 20 topics, we observed mergers of clearly distinguishable topics which were distinct in models with higher numbers of topics. For example, the topics Therapy and Livelihood were observed together in a single topic with top ranked words including “work, job, psych, health”.

In the second phase of the LDA analysis, we incorporated the quantitative measures of each topic’s coherence [31] and specificity (distance to corpus score) [32] provided by the Mallet software diagnostic output. We used these in models of 20, 25 and 30 topics to flag topics which initially seemed substantive, but which were possibly incoherent or similar to a representation of the entire corpus.

In the third and final phase of the LDA analysis, we used domain knowledge of the context in which the topics occurred most frequently and the posts in which the topics were highly prevalent to validate their nature. Based on this combination of qualitative, quantitative and domain knowledge, we concluded that a model with 25 topics was the most intelligible. However, there were four ‘junk’ topics among these for which a meaningful interpretation could not be identified. This included two topics which had low coherence scores relative to the other topics (eg, “back head pain put water body eyes cold cat front”) and two topics which had low specificity (distance to corpus scores) (eg, “back told time thought home felt day friend wanted asked”), although coherence and specificity scores were not used as executive criteria for exclusion. After excluding the four meaningless topics, the final model included 21 topics. Labels were assigned to each topic by BC with the assistance of the MHISG manager (JR) who provided domain expertise based on the overarching concept apparent in each topic’s word list and by perusing posts that were comprised predominantly of each single topic.

### Part 2: Comparison of topic and sentiment expression across user groups

Users were divided into “super users” (the top 1 % of users by posting frequency) and “other users” (the remaining 99 % of users). As shown in Table 1, the 29 super users contributed just under three times as many posts in total as the 2903 other users, but their individual posts contained significantly fewer words (a difference of 35 words between medians; Mann–Whitney U = 1.23 x 10^9^, *p* < .001). In total, other users initiated more than twice as many threads as super users. Super users contributed more than four times as many posts to the threads that were initiated by fellow super users than to the threads of other users. Other users contributed three times as many posts to threads initiated by fellow other users than to threads of super users.

**Table 1:** User groups

	Super users (*n* = 29)	Other users (*n* = 2903)
Total number of posts	96,896	34,108
Total threads initiated	2133	4607
Posts in super user threads	79,584	8,358
Posts in other user threads	17,312	25,750
Mean (sd) post word count	70 (114)	110 (143)
Median post word count	35	67

To determine the nature of the content contributed by super users we conducted two chi-square tests for each of the 21 topics. In the first, we analysed all posts, comparing the odds that the two different user group’s posts contained content from each topic. In the second, we analysed only super user posts. We similarly compared the topic-specific odds of posts written by super users in response to super users with those that super users wrote in response to other users. We used a Bonferroni correction to adjust for multiple comparisons (n = 42). Thus a value of *p* < .001 was required for statistical significance.

## Results

### Part 1: Identification of topics

The 10 most frequently occurring words in each of the 21 topics produced by the LDA analysis are shown in Table 2. The topic comprising the largest percentage of the corpus was Social Relations, making up 8.21 % of all words in the collection (excluding stop words).

**Table 2:** The 10 most frequently occurring words in each topic

Topic Label	Proportion of corpus (%)	Top 10 most frequent words
Social Relations	8.21	people person make feel good time understand life relationship wrong
Depression	7.20	depression feel people friends time years life anxiety work year
Anxiety	7.09	feel feeling anxiety bad time hard head thoughts sick day
Recovery Journey	6.11	life love feel pain world heart time hope find day
Daily Functioning	5.09	day sleep night today good work bed time morning tomorrow
Affection	5.01	hugs hope big good love thinking xxx hear hug happy
Family	4.83	family daughter kids mum mother son husband parents time children
Therapy	4.56	health mental good support psychologist therapy find talk psych psychiatrist
Medication	4.32	meds medication anxiety taking side depression effects weeks doctor dose
Mental Illness	4.13	people mental illness bipolar good depression disorder life problems important
BlueBoard	4.03	post read thread write writing posts squad mod people reading
Positive Change	3.34	life time years happy great love wonderful world part important
Co-created Fiction	2.85	[username]^a^ eyes back [username]^a^ head hand face tears water ship
Livelihood	2.79	money work job people pay Centrelink^b^ government system Australia health
Chat	2.72	lol beer dog cool awesome love gonna yeah [username]^a^ [username]^a^
Parenting role	2.63	kids bit [username]^a^ time school life stuff kinda mum daughter
Bipolar	2.50	bipolar depression mood disorder manic diagnosed meds normal mania diagnosis
Philosophy	1.97	world brain mind people god human women science society power
Entertainment	1.61	music love song play movie good watch book playing songs
Food and Drink	1.58	eat food tea eating water chocolate coffee good chicken drink
Drugs and Alcohol	1.14	alcohol drink drinking smoking shit drugs smoke drug stop weed

^a^In accordance with the study ethics protocol usernames have been omitted

^b^Centrelink is an Australian Government organisation which provides social services and welfare payments

The distinctiveness of several topics was apparent directly from the word lists output by the LDA analysis. Their nature was evident from the presence of multiple words with a common theme. These topics were Medication, Therapy, Livelihood, Entertainment, Family, BlueBoard, Food and Drink, Affection, Bipolar, Anxiety, and Drugs and Alcohol. Table 3 presents a short quotation from the corpus to illustrate each of these topics.

**Table 3:** Quotations exemplifying the nature of each topic

Topic	Exemplary quotations
Social Relations	“I can see now that I have been in controlling relationships but I'm not weak enough to just accept it & not strong enough to stand up for myself enough.” “In life friends come and go, its the natural order, and if people are in a better place then it is a good thing.”
Depression	“Um, hi. I'm new to this website, so I just wanted to share a bit about myself. I've dealt with depression for almost four years of my life now (I am 18) and also have social anxiety. I have no one to talk to in my life because most of my friends are all too busy for me apparently… Anyway, I could go on forever about my depression and my story, but I won't bore you guys.. (just yet;)
Anxiety	“I've always struggled with stress & anxiety, and have had mild panic attacks in the past. But what I have tended to do lately is be 'triggered' by something, then get really emotional, cry, stress out, non stop thoughts, at time weird breathing etc.”
Recovery Journey	“I am strong because I am weak. I am beautiful because I know my flaws. I am a lover because I'm a fighter. I am fearless because I have been afraid. I am wise because I have been foolish… And I can laugh because I've known sadness” “Hi [username] My thoughts and feelings are very much the same, but i was put on this earth for a reason and I'm sure it was not to suffer for my whole life, there is happiness out there I believe that otherwise what is the point of being here”
Daily Functioning	“Hi guys, Had a pretty lame day today. Because I got so little sleep last night (~3 h), I ended up getting my daughter an extra day at childcare. Then I was supposed to be productive, but got almost nothing done all day. My anxiety has really “re-generalised” lately… Will see how I go with work tomorrow… I'm not feeling too bad really despite today… Just feeling tired… More sleep tonight will help”
Affection	“Hi sweetie, Big hugs to you, sounds like a hard day Know what, I don't think we've been introduced…I'm [username], welcome. Can't imagine how tough it would be, hang in there, sending hugs and happy vibes your way.”
Family	“It pains me to read about families that do not support each other! I realize i have been fortunate to be raised by loving parents and be able to become a good parent to my now grown children. I love to see my children support each other and continue the close bonds that have formed over the years. I wish this was possible for all families.”
Therapy	“I really find it hard to talk in therapy. I just feel that the therapist does not feel the same way as I do so there is no way he can relate. That is why I decided to come here.”
Medication	“Hi I have anxiety (OCDs) and depression. I am currently on several medications - 1 week ago I increased Zoloft (Sertraline) to 200 mg, prior to that 50 mg - 150 mg over 4 weeks. My question is has anyone on BB had experience with Zoloft and know how long it takes to kick in?”
Mental Illness	“Reading as you do, [username], will help you on your journey. I have been doing what you are doing now for many years. I have changed my mind many times and considered the biological, environmental and psychological forces of what we call bipolar. “Dear [username], you are right. People with a mental illness are under represented in research. Not many of us are able to do research on mental illness. There are many reasons for this”
BlueBoard	“Hey [username] :) Great to see you posting again. To find the thread about new avatars, go to the Blueboard Notices forum, Sub-forum Blueboard Notices, thread titled Image and Photo Posting. The last post on that thread explains how to upload custom avatars.”
Positive Change	“I am deeply proud to be a member of this wonderfully diverse community of individuals, linked by mental health issues and yet so different in life circumstances. We all have so much to offer each other by sharing our lives, our trials and triumphs. I am a better person for having the good fortune of being introduced to you all through BlueBoard. Humbly and wholly, I offer up my thanks to you all” “Truth be told, my friend, I would not want you to take away my pain. It is an important motivator in my life, an important process. I do not identify myself through this pain, but it acts as a catalyst for change in my life. From this great perceived negative, I am reborn into the positive”
Co-created Fiction	“[username] gives out an audible cry of fear, [username] is instantly at her side, arm around her, dagger in hand. She hugs [username], eyes fixed on the flaming bird of destruction which is almost upon them. The crew shouts increase as they prepare the cannons and water down the ship” “Tears fall onto her lap like a waterfall, she hides her face in her hands sinking back to the wet wood soaked from her tears.”
Livelihood	“Hi [username], if you have some financial trouble and need some money to fix your car there are government organization who can lend you some interest free money.”
Chat	“We should have beer garden day. lol everyone sitting around in their backyard beer gardens or out at beer gardens” “[username] is BACK!!!!!!! Woooooooo hoooooooooooo!!!! :D :D :D Hasn't been the same without you! This calls for an undies-on-head dance! [username]!!! Get thee to the Beer Garden STAT!!!
Parenting role	“School excursion tomorrow. daughter is sooooooooo excited. oh crap- i didnt organise everything- usually i do that on Thursday- but i should have done that tonight- after school snack, footy stuff and piano stuff as well as breakkie and getting kids ready.” “Im sure those routines will need adjusting etc.- but its kinda what we do, try to do or what we want to do. so… thinking a bit of time management might make it all happen more. and it fits around after school things”
Bipolar	“For me it varies quite a lot. I can go lengthy periods where everything is fine and episodes are infrequent though long when they do hit. At other times, I cycle more rapidly and can go from depression to mania with no balanced state in between.”
Philosophy	“Physics postulates that there are an infinite number of parallel universes in existence, all of them either subtly or vastly different from each other. Psychology and Philosophy confirm this, every one of us is a parallel universe of subjective reality, uniquely coexisting with the others.” “In it's simplest form, this is kind of my thesis: In societies dominated by patriarchal attitudes, cautionary tales often depict powerful women as physically and emotionally ugly… which greatly disadvantages women in society striving for power”
Entertainment	“The song in free to listen to on uT on the net. Most of his songs are. Tony Joe White is one of my favorite singers.”
Food and Drink	“I was wondering if anyone here enjoys cooking? I am trying to make meals which have more vegetables and healthy foods”
Drugs and Alcohol	“im so blerrrrr, spent so much, trying to keep the boredom away too much boose too much smoke just to get myself out of bed i had to dilute myself, deression is hell”

The nature of other topics was more apparent once the words were contextualised by perusing posts for which the topic made up the majority of the content and by incorporating the domain expertise of the BlueBoard manager. Table 3 presents quotations that provide the context for interpreting these topics.

Three topics (Parenting Role, Co-created Fiction and Chat) almost exclusively involved super users. The usernames that were included in the word lists for these topics were those of the super users who were known to regularly engage in conversations on these topics. Parenting Role largely comprised conversation about managing one’s parental responsibilities while also managing one’s mental illness. Co-created Fiction comprised words and usernames which were frequently included in stories narrated in the third person by a particular sub-group of super users. The stories were imaginary journeys in which the users underwent heroic challenges and supported each other. Chat comprised words that were highly typical of conversation in the largest thread on BlueBoard – “The Beer Garden”. This was a user-established thread which users visited for companionship and to socialise.

The topic Mental Illness comprised meta-level content referring to mental illness in general terms. It differed from the topics Bipolar, Anxiety and Depression which comprised content which was more specifically about the individual’s personal experience of these illnesses. The topic Depression was particularly exemplary of this, comprising many words that were not semantically related, but which were experientially related to depression. The topic Depression had the lowest specificity (distance to corpus score) of all the topics, signifying that of all the topics it was the most similar to a random selection of words from the whole corpus. The most frequently occurring word was depression itself. However, based on the other words in the list, it appears that the topic comprised a broader notion of depression than the clinical definition, encompassing themes of dysphoria and social isolation which may be common among people seeking social support.

### Part 2: Differences between user groups in frequency of topic expression

**Fig. 1:**
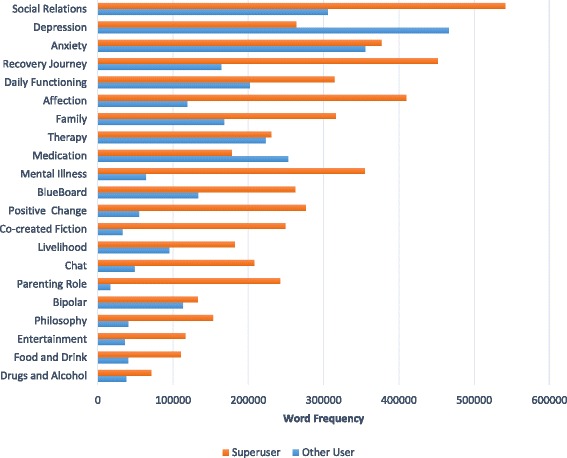
Total frequency of words written on each topic by super users and other users
  To investigate the relative inclinations of users to write content on each of these topics, we compared the odds that their content was ‘on’ versus ‘not on’ each topic. The results are shown in Table 4. Super users wrote relatively less than other users on seven topics: Depression, Medication, Therapy, Anxiety, Bipolar, Daily Functioning and Social Relations. For example, the odds that super users incorporated content containing words from the topic Depression were one-quarter that of the odds that other users incorporated such content. Conversely, the odds that super users wrote content were significantly higher for 13 topics. These were Parenting Role, Co-created Fiction, Mental Illness, Positive Change, Chat, Philosophy, Affection, Entertainment, Recovery Journey, Food and Drink, BlueBoard, Livelihood, and Family. The largest discrepancy was for the topic Parenting Role, with the odds of super users posting on this topic being 7.97 times that for other users. There was not a significant difference in posting by the two user groups for the topic Drugs and Alcohol.

(i) Super user vs other users: across all posts
  The total number of words written by each user group on each topic is shown in Fig. 1. It can be seen that for all topics other than Depression and Medication, super users wrote more content than other users.

**Table 4:** Odds ratios and chi-square analyses for super user vs other user content for each topic

Topic	Chi Square	Odds Ratio (95 % CI)	*P*
Depression	284428.58	0.27 (0.27–0.28)	<0.0001
Medication	108165.68	0.36 (0.36–0.37)	<0.0001
Therapy	40040.97	0.55 (0.54–0.55)	<0.0001
Anxiety	60723.13	0.55 (0.55–0.55)	<0.0001
Bipolar	12566.17	0.63 (0.63–0.64)	<0.0001
Daily Functioning	3423.64	0.84 (0.84–0.85)	<0.0001
Social Relations	239.74	0.96 (0.96–0.97)	<0.0001
Drugs And Alcohol	4.52	1.01 (1.00–1.03)	0.03
Family	87.00	1.03 (1.02–1.04)	<0.0001
Livelihood	147.06	1.05 (1.04–1.06)	<0.0001
Blueboard	460.79	1.08 (1.07–1.08)	<0.0001
Food And Drink	4755.95	1.49 (1.47–1.51)	<0.0001
Recovery Journey	21845.44	1.55 (1.54–1.56)	<0.0001
Entertainment	8999.79	1.76 (1.74–1.78)	<0.0001
Affection	40184.47	1.94 (1.93–1.96)	<0.0001
Philosophy	17719.14	2.08 (2.05–2.10)	<0.0001
Chat	30423.66	2.35 (2.33–2.38)	<0.0001
Positive Change	52598.04	2.82 (2.79–2.84)	<0.0001
Mental Illness	76564.00	3.13 (3.11–3.16)	<0.0001
Co-Created Fiction	70981.96	4.22 (4.17–4.27)	<0.0001
Parenting Role	96582.60	7.97 (7.85–8.10)	<0.0001
(ii) Super user responses to super user vs other user threads (posts by super users only)
  The number of words written by super users on each topic in response to fellow super users compared with responses to other users is shown in Fig. 2. On each topic, super users wrote more content in response to fellow super users than other users.

**Fig. 2:**
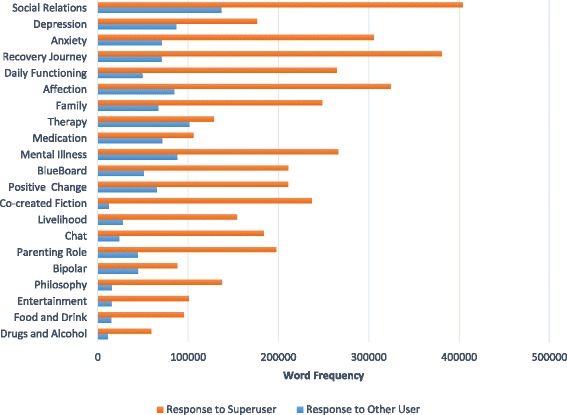
Topic-specific word frequency in posts by super users responding to super users vs other users
  Results of chi square tests comparing the odds of response for the two types of responses are shown in Table 5 for each topic. Odds were significantly lower for responses to super users compared to responses to other users for seven topics: Therapy, Medication, Bipolar, Depression, Social Relations, Mental Illness, and Positive Change. The largest discrepancy was for Therapy, which showed that super users were one-third as likely to write about therapy in response to super users compared to other users. The odds that responses to super users contained more content were significantly higher for 13 topics. These were Co-created Fiction, Philosophy, Chat, Entertainment, Food and Drink, Livelihood, Recovery Journey, Daily Functioning, Drugs and Alcohol. Parenting Role, Anxiety, BlueBoard, and Affection. The largest discrepancy was for the topic Parenting Role, with super users being 5.29 times as likely to write about this content in response to super users than in response to other users. There was not a significant difference for the topic Family.

**Table 5:** Odds ratios and chi-square analyses comparing super user responses to super users vs other users across topics

Topic	Chi Square	Odds Ratio (95 % CI)	*p*
Therapy	74299.69	0.32 (0.32–0.33)	< .0001
Medication	39546.97	0.39 (0.38–0.39)	< .0001
Bipolar	12380.59	0.53 (0.52–0.53)	< .0001
Depression	22647.52	0.53 (0.53–0.54)	< .0001
Social Relations	5770.87	0.78 (0.77–0.78)	< .0001
Mental Illness	2961.97	0.80 (0.80–0.81)	< .0001
Positive Change	1026.92	0.86 (0.86–0.87)	< .0001
Family	0.75	1.00 (1.00–1.01)	0.39
Affection	70.18	1.03 (1.03–1.04)	< .0001
Blueboard	522.38	1.12 (1.11–1.13)	< .0001
Anxiety	1417.75	1.18 (1.17–1.19)	< .0001
Parenting Role	1304.21	1.21 (1.20–1.23)	< .0001
Drugs And Alcohol	1105.30	1.40 (1.37–1.43)	< .0001
Daily Functioning	5892.94	1.46 (1.45–1.48)	< .0001
Recovery Journey	9230.50	1.50 (1.49–1.51)	< .0001
Livelihood	3861.81	1.50 (1.48–1.52)	< .0001
Food And Drink	3687.71	1.69 (1.66–1.72)	< .0001
Entertainment	4388.67	1.76 (1.73–1.79)	< .0001
Chat	12012.34	2.09 (2.07–2.12)	< .0001
Philosophy	11360.35	2.39 (2.35–2.43)	< .0001
Co-Created Fiction	41183.62	5.29 (5.19–5.38)	< .0001

## Discussion

### Principal findings

The current study used a computer-assisted method to identify topics in a mental health Internet support group. In particular, an analysis using LDA enabled us objectively to identify 21 topics which constituted the major components of discussion on the mental health ISG BlueBoard. There were significant differences in the frequency with which highly engaged super users wrote content on these topics compared to other users and between the content of super user posts in response to posts from users of each group. The pattern of results was consistent with a model that suggests more highly engaged users play a role as active help providers, particularly with respect to the provision of companionship and emotional support, but are relatively less inclined than other users to write content about clinical topics such as medication and treatment.

### Part 1: Identification of topics

The topics identified in the current study comprised meaningful themes with relevance to mental health. The most frequently discussed topic was Social Relations, followed by two condition-specific topics, Depression and Anxiety. Bipolar was also identified as a topic. These three condition-specific topics are also the subject of disorder specific sub-forums on BlueBoard, e.g. “Living with Depression”, which contain the highest number of posts of all disorder specific sub-forums. This lends some support to the validity of the LDA analysis. It is important to note that these topics and sub-forums are not redundant categorisations. This is because BlueBoard sub-forums often contain content that does not pertain specifically to a single disorder. For example, content about experience of comorbid anxiety and depression may be found in either or any forum. The LDA topics are able to distinguish between these types of content, regardless of their location in the organisational structure of BlueBoard.

From the perspective of the framework proposed by Preece [19], some of the identified topics may be classified as ‘off-topic’, for example Chat, whereas other topics may be classified as ‘on-topic’, for example Therapy. This framework is limited in that it fails to consider the relevance of ‘off-topic’ content within the context of its broader utility. The findings of the current study are more consistent with frameworks which classify different types of content into categories of peer-support. From this perspective, the topic Chat may be considered relevant to companionship, and the topic Therapy may be considered relevant to informational support. Thus, although Chat is not directly related to mental health, its relevance in the context of peer-support is apparent.

There are clear links between the topics identified by automated processing in the current study and the different types of peer-support content that have previously been defined in the social-support literature [33] and implemented in content analyses of MHISGs [15]. In particular, the topics identified in the current study were consistent with the social-support categories of disclosure, experiential knowledge, information support, companionship, emotional support, group structure and process, and cognitive guidance. Topics involving specific types of mental health issues including Depression, Anxiety, Bipolar, and Drugs and Alcohol, comprised users’ first and second hand experiences. Thus, they could be seen to be related to ‘disclosure’ and ‘experiential knowledge’; e.g. “hi. I'm new to this website, so I just wanted to share a bit about myself. I've dealt with depression for almost four years of my life now”. Topics involving circumstances and contexts in which mental health issues occur and impact, including Daily Functioning, Social Relations, Livelihood, Family, Food and Drink, and Parenting Role, also fit disclosure and experiential knowledge categorisations; e.g. “I become so depressed I cant get out of bed, dont eat, cry all the time..... I just cant function” . The topics Medication and Therapy may also fit these categorisations but given their direct role in treatment, it is likely that their primary relevance is to the peer-support category of ‘information support’; e.g. “I've been using lamictal and epilium for a number of years, it's the best combination I've come across.”. The topics Chat, Entertainment and Philosophy involved conversational matters typical of ‘companionship’; e.g. “I may be feeling low…but this gave me a laugh, it's my type of humor exactly…” . The topic Affection related to care and concern for others, factors that are typical of ‘emotional support’; e.g. “Can't imagine how tough it would be, hang in there, sending hugs and happy vibes your way.”. The topic Co-created Fiction included elements of both companionship and emotional support; e.g. ““[username] gives out an audible cry of fear, [username] is instantly at her side, arm around her, dagger in hand.”. Finally, the topic BlueBoard involved references to the forum itself and thus concerned references to ‘group structure and process’; e.g. “Not sure if you read post #116 in this thread - we posted stuff at the same time, so you may have missed it.”.

The remaining topics, Positive Change, Recovery Journey and Mental Illness are consistent with ‘cognitive guidance’, though not necessarily in the form of direct advice. All three topics included the word “life” and it is clear from our perusal of typical posts that the content explores the impact of mental illness in their life and the experience of finding a pathway to recovery; e.g. “I know many people with bipolar who are happy with their life no matter what problems they face. Yet what our world teaches people with mental illness is that they are unlucky and that their life will be a troubled one”. This content is consistent with the consumer/carer model of recovery which emphasises personally meaningful elements of recovery such as hope, healing, empowerment and connection [34]. This contrasts with the traditional clinical model which focuses on the efficacy of treatments in reducing the symptoms that formally define mental illnesses. This clinical symptom-focussed approach is more apparent in topics which focus directly on illnesses and treatments e.g. Bipolar and Medication.

We highlight the above associations between the LDA topics and social support content for the purpose of describing trends observed in the data across user groups, which are discussed below. We acknowledge these above associations and below trends represent our interpretation of the data and that this interpretation is subjective. However, our methodology has largely deferred the point at which subjective interpretation enters the study until after the computation of the results (with the exception of the number of topics selected). Thus the strength of this analysis is not only in the novel perspective it provides on quantitative large-scale trends in the data, but it is also inherent in the transparency and replicability of the analysis. A content analysis of this nature has not previously been conducted on a MHISG.

### Part 2: Differences between user groups

Overall, compared to other board users, super users were relatively more engaged in topics which related to companionship, emotional support and cognitive restructuring with a focus on consumer/carer defined recovery. This was the case for 10 out of the 13 topics for which super users wrote relatively more content than other users. In contrast, other users were relatively more engaged in topics which related to disclosure, experiential knowledge and informational support. This was the case for all seven of the topics for which other users wrote relatively more content than super users. This suggests a greater focus by other users on the traditional clinical symptom-focused approach to recovery.

Although super users have been known to identify themselves as ‘active help providers’ [14], the current study raises the question of whether the type of support provided by these users matches the type of support being sought. The findings of the current study suggest that there is an overall discrepancy in the type of content in which the two user groups prefer to engage. If this difference is the consequence of a discrepancy in perceptions of recovery, super users may be well placed to support fellow super users, but not necessarily be the best placed to provide the support being sought by other users. However, it is also evident that super users change the nature of their content depending on type of user to whom they are responding. Super user responses in other users’ threads were found to have higher odds for 5 of the 7 topics in which other users were previously observed to be relatively more engaged, and in particular in topics that were typical of experiential knowledge and informational support such as depression and medication. This suggests that super users actively change the type of content they contribute to align with the interests of other users when they are responding to them. This is consistent with the idea that super users are generally ‘active help providers’. However, we acknowledge that there may be other explanations for the data and that responsiveness to communication context does not necessarily imply that the super user is delivering help. For example, based on the current data, we cannot exclude the possibility that they are seeking or receiving help. Further research is required to investigate this issue.

A previous qualitative investigation of BlueBoard that examined depression information needs [35], found that ‘coping with depression’ (in particular, symptoms) was the most frequently explicitly and implicitly stated information need in user posts. This finding appears consistent with the type of content most often posted by other users in the current study. Furthermore, in another study of BlueBoard which involved a qualitative analysis of user-perceived advantages of participation in the board, the two most frequently cited types of advantage were (i) positive personal change, encompassing emotions such as: feeling glad, grateful, hopeful and inspired; cognitive effects, including changing the way a person thought about an issue; and behavioural effects, including choosing to see a doctor, and (ii) positive social interactions and support [36]. Few posts referred to symptom or disorder-specific advantages and it was concluded that the benefits of forum participation may be best conceptualised in terms of supporting overall recovery rather than as disorder or symptom-specific effects. In concert with the findings of the current study, this may suggest that the input provided by super users is consistent with the type of support that is valued by members, and is also consistent with broader evidence regarding the benefits of participating in ISGs, including increased sense of empowerment [7, 9], and improved perceived quality of life [9] and self-esteem [9].

### Limitations and future research

The current research has three main limitations. The frequency with which topics are expressed in a MHISG was represented in the current study in both absolute and relative terms. However, such data does not provide insight into the subjective experience of the user reading the topic content. For example, a post may contain a short statement about medication followed by a long story about a person’s experience with depression. The reference to the medication may be of great importance in the story, but due to the associated small word count the subjectively important status is not represented in the data. Thus the current research methodology can provide a broad overview of the nature of the content in a MHISG, but is limited in the extent to which it represents a user’s intention or another user’s interpretation of the content. Future research seeking to address this may incorporate qualitative and or supervised machine learning methods to the analysis [37].

The second limitation of the study is that the dichotomisation of users according to their posting frequency (super user vs other users) provides a limited framework for defining the nature of a user’s participation in an ISG. The role performed by a user can be classified by more nuanced metrics [16] and may change over time [38]. However, posting frequency is the most common way that research has classified users to date [16], often with an assumption that users with higher frequencies of posts contribute greater value to the ISG. The current study demonstrates broadly that these users contribute *different* value. Future research may demonstrate further differentiation in the kinds of value contributed by different users. In non-mental health contexts, researchers have focused on differentiating providers and seekers of emotional support, information support and companionship in ISGs [38–40]. In a MHISG context there may be other important roles such as users whose posts facilitate a decrease in self-stigma or users who are effective in supporting other users who are in crisis to seek professional care.

Lastly, it is both a strength and limitation of this study that it was focused on a MHISG in which moderators do not play an active role in the community. Consequently the results may not generalise directly to differently constituted MHISGs. However the findings are strengthened by the fact that the behaviour we have observed occurred without the potentially biasing influence of ISG staff. Further research is required to understand how this may vary in different MHISGs

## Conclusion

The current study demonstrates the utility of a computational method for analysing the content of MHSIGs. This technique enables trends in user engagement patterns to be investigated objectively and on a large scale. The pattern of findings in the current study has provided support for the notion that the most active members in a MHISG are generally ‘active help providers’. The findings suggest that super users serve the role of emotionally supportive companions with a focus on topics broadly resembling the consumer/carer model of recovery. Other users engage in topics with a greater focus on experiential knowledge, disclosure, and informational support, a pattern resembling the clinical symptom-focussed approach to recovery. However, super users also modify their content to be more like that of other users when responding to them. These findings highlight similarities between the nature of super user engagement and existing evidence regarding the therapeutic outcome of user participation in ISGs, suggesting that the most highly engaged users may play an important role in this outcome.

## Abbreviations

ISG: Internet support group
LDA: Latent Dirichlet Allocation
MHISG: Mental health internet support group
